# Cost-Effectiveness of Preventive Interventions to Reduce Alcohol Consumption in Denmark

**DOI:** 10.1371/journal.pone.0088041

**Published:** 2014-02-05

**Authors:** Astrid Ledgaard Holm, Lennert Veerman, Linda Cobiac, Ola Ekholm, Finn Diderichsen

**Affiliations:** 1 Department of Public Health, University of Copenhagen, Copenhagen, Denmark; 2 School of Population Health, The University of Queensland, Brisbane, Australia; 3 National Institute of Public Health, University of Southern Denmark, Copenhagen, Denmark; Erasmus University Rotterdam, Netherlands

## Abstract

**Introduction:**

Excessive alcohol consumption increases the risk of many diseases and injuries, and the Global Burden of Disease 2010 study estimated that 6% of the burden of disease in Denmark is due to alcohol consumption. Alcohol consumption thus places a considerable economic burden on society.

**Methods:**

We analysed the cost-effectiveness of six interventions aimed at preventing alcohol abuse in the adult Danish population: 30% increased taxation, increased minimum legal drinking age, advertisement bans, limited hours of retail sales, and brief and longer individual interventions. Potential health effects were evaluated as changes in incidence, prevalence and mortality of alcohol-related diseases and injuries. Net costs were calculated as the sum of intervention costs and cost offsets related to treatment of alcohol-related outcomes, based on health care costs from Danish national registers. Cost-effectiveness was evaluated by calculating incremental cost-effectiveness ratios (ICERs) for each intervention. We also created an intervention pathway to determine the optimal sequence of interventions and their combined effects.

**Results:**

Three of the analysed interventions (advertising bans, limited hours of retail sales and taxation) were cost-saving, and the remaining three interventions were all cost-effective. Net costs varied from € -17 million per year for advertisement ban to € 8 million for longer individual intervention. Effectiveness varied from 115 disability-adjusted life years (DALY) per year for minimum legal drinking age to 2,900 DALY for advertisement ban. The total annual effect if all interventions were implemented would be 7,300 DALY, with a net cost of € -30 million.

**Conclusion:**

Our results show that interventions targeting the whole population were more effective than individual-focused interventions. A ban on alcohol advertising, limited hours of retail sale and increased taxation had the highest probability of being cost-saving and should thus be first priority for implementation.

## Introduction

Alcohol consumption is widespread in Denmark. Alcohol is associated with times of celebration, but for many Danes alcohol consumption is not restricted to this. On average every Dane over the age of 14 consumed 11.3 litres of pure alcohol in 2010[1].

Excess consumption of alcohol increases the risk of stroke and hypertensive heart disease, gastro intestinal disorders such as pancreatitis and cirrhosis, several types of cancers and injuries[2]–[8]. However, there is evidence that moderate consumption of alcohol has a protective effect on ischaemic heart disease[9].

Consumption of moderate amounts of alcohol is therefore advised. In Denmark the guidelines from the Health and Medicines Authority states that low risk alcohol consumption is less than 7 standard drinks (a Danish standard drink is equivalent to 12 grams pure alcohol[10]) per week for women and 14 standard drinks per week for men, whereas alcohol consumption of more than 14 standard drinks per week for women and 21 standard drinks per week for men involves a high risk of alcohol-related diseases[10].

Alcohol consumption has a large impact on the burden of disease, and places a considerable economic burden on societies. According to the new estimates from the Global Burden of Disease 2010 study, about 6% of the burden of disease in Denmark is due to alcohol consumption[11], and 5% of all deaths, corresponding to more than 3,000 deaths per year, are alcohol-related[12]. Further, it has been estimated that people exceeding the recommended maximum drinking limits die on average of 4–5 years prematurely[12]. Economically, excess alcohol consumption has been estimated to cost society more than 1% of the gross national product in high- and middle-income countries[13]. Further, a Danish analysis of the socio-economic consequences of alcohol consumption estimated the overall costs of alcohol consumption to be between € 160 million using the friction cost method and € 1.1 billion using the human capital method (population approx. 5.6 million)[12].

It is therefore important to design and implement interventions, which can reduce population alcohol consumption and thereby reduce prevalence of alcohol-related diseases and injuries. Cost-effectiveness analysis can be used to evaluate the potential costs and health effects of implementing interventions, compared with current practice, other possible interventions or doing nothing. Cost-effectiveness analysis can thus be a useful tool for decision-making and resource allocation[14], [15].

Studies have analyzed the cost-effectiveness of alcohol interventions[16]–[18], but due to contextual differences the results are difficult to transfer between settings. A generalized approach to cost-effectiveness has been proposed[19] and although this approach has been applied to alcohol interventions[20], limitations regarding external validity hinder the transferability of the results to a Danish context[21].

In our study, we estimate the cost-effectiveness of six interventions aimed at preventing the burden of disease from alcohol-related diseases and injuries in Denmark, through reduced consumption of alcohol. Each intervention is analysed separately and the most cost-effective options are then combined to determine the optimal intervention mix. In the analyses we make use of the unique Danish registers for information on disease incidence, mortality and costs for all individuals in the Danish population.

## Methods

### Interventions

The following six interventions were included in the analyses. The interventions were selected from relevant alcohol interventions based on availability of sufficient evidence to support a cost-effectiveness analysis. All interventions focused on general alcohol prevention in the adult population aged 16 years or older.

1. **30% in taxation of alcoholic beverages.** In Denmark alcohol taxes vary according to beverage type, with heavier taxation on spirits than wine and beer. In 2009 the tax on beer, wine and spirits was € 6.8, € 6.7 and € 20.1 respectively per litre of pure alcohol, or € 0.1 per 33cl bottle or can of beer, € 0.6 per 75cl bottle of wine and € 5.6 per 70cl bottle of spirits. We modelled the effects of a 30% increase in the current alcohol taxation.
2. **Increased minimum legal drinking age.** The minimum legal age for consuming alcohol in bars, restaurants, etc. is 18 years in Denmark, but the minimum legal age for purchasing alcohol in retail outlets is only 16 years. The effect of an overall minimum legal drinking age of 18 years was modelled.
3. **Advertising bans.** Currently, only alcohol advertisement targeted at children is illegal. We analyzed the effects of a comprehensive ban on alcohol advertisement (via billboards, television, radio, etc.).
4. **Limited hours of alcohol retail sales.** With the current Danish legislation, it is legal for retail outlets to sell alcohol to take home, in their opening hours, without licensing; a license is only required to serve alcohol on the premises. Legislation restricting the hours of sale of alcohol has been proposed as a means of limiting alcohol purchase. Licenses are issued and managed by the municipalities and local councils.
5. **Brief interventions by telephone.** According to Danish legislation, municipalities are responsible for preventing alcohol misuse and providing treatment of alcohol abuse to their citizens. We modelled the effects of brief (15 min.) consultations, providing information and support, conducted by trained staff over the phone.
6. **Longer intervention offered in municipal prevention centres.** Up to five one-hour consultations offered to citizens with hazardous or harmful alcohol consumption levels. Conducted face-to-face by trained staff in municipal prevention centres.

Input parameters for each intervention are shown in table 1, and described in appendix S1. The target group of each intervention was determined by the age groups targeted and the baseline level of alcohol consumption. Intervention effects on alcohol consumption were based on evidence from the alcohol literature, except for the minimum legal drinking age intervention, where mean effect is calculated based on the difference between hazardous and harmful consumption in the target group (see appendix S1). Effects were measured as relative or absolute change in grams of alcohol consumed per day. In order to increase comparability between interventions, effects were evaluated for one year at full implementation, thereafter assuming a decay of 100% for interventions targeting the entire population and 50% for individually targeted interventions. Intervention costs were estimated from a Danish health sector perspective, and total costs included government costs associated with delivery and enforcement of interventions and costs of materials. Costs associated with lost productivity, time costs for patients due to participation in interventions or costs to others than the patient (e.g. family) were excluded. Interventions were analysed as operating ready, which means that costs associated with research and development of interventions were not included.

**Table 1:** Intervention parameters (modelling methods, assumptions and data sources are described in appendixs S1 and S2).

Intervention	Target group	Proportion of population	Mean effect in target group	Decay rate	Mean cost (€ million)
1. 30% taxation	Whole population	100%	−6.9%	100%	€ 0^a^
2. Minimum legal drinking age	Population aged 16–17 years	12%	−32.8 g/day for males −26.1 g/day for females	100%	€ 375,000 yearly costs + € 270,000 in year 1
3. Advertising bans	Whole population	100%	−4%	100%	€ 100,000 yearly costs + € 270,000 in year 1
4. Reduced retail opening hours	Whole population	100%	−3%	100%	€ 375,000 yearly costs + € 270,000 in year 1
5. Brief intervention	Hazardous/harmful drinkers	3%	−5.4 g/day	50%	€ 2.2 m yearly costs
6. Longer intervention	Hazardous/harmful drinkers	1%	−5.4 g/day	50%	€ 8.9 m yearly costs

a The Danish Ministry of Taxation estimates that current costs will not change with an increased taxation level[35]. Increased taxation is therefore assumed to be cost neutral.

### Current alcohol consumption

Data on current levels of alcohol consumption were taken from the Danish Health and Morbidity Survey (the national sample in the Danish National Health Survey)[22]. Underreporting of alcohol consumption is often seen in surveys compared to sales statistics[23], [24], and we therefore adjusted the alcohol consumption data for underreporting, using sales statistics[25]. Based on the guidelines from the Danish Health and Medicines Authority[10], four categories of alcohol consumption were created. Age-specific population distributions were calculated for these categories. Appendix S2 describes further details on the adjustment and analyses of alcohol consumption data.

### Health effects and net costs

Potential health effects of each of the included interventions were evaluated by determining changes in incidence, prevalence and mortality of alcohol-related diseases and injuries, using a multiple cohort, multi-state life table approach[26]. Diseases included in the analyses were ischaemic heart disease, ischaemic and hemorrhagic stroke, hypertensive heart disease, pancreatitis, cirrhosis, cancer of the breast (in women), mouth and oropharynx, oesophagus, liver, larynx, colon and rectum, and injuries caused by road traffic accidents (RTA) or other accidents (non-RTA). Data on disease incidence and mortality were taken from The Danish National Patient Register[27] and The Danish Register of Causes of Death[28], both of which cover the entire Danish population and can be linked for all individuals. Health outcomes were measured in disability-adjusted life years (DALYs)[29].

Net costs of each intervention were calculated as the sum of intervention costs and costs of prevented health care utilisation (cost offsets) related to treatment of alcohol-related diseases and injuries. Cost offsets were estimated using multiple regression analyses, based on health care system costs (including primary and secondary sector and medicine costs) from Danish national registers[30] and data on municipal health care costs from the Municipality of Copenhagen[unpublished data]. Costs were derived for each modelled disease, based on rates of disease in 2009. Average health care costs due to diseases not associated with alcohol consumption were included in the analysis in order to account for costs in added years of life. For further information regarding the modelling methods see appendix S2.

### Cost-effectiveness analyses

The cost-effectiveness analyses used the year 2009 as baseline, and all cost offsets and health effects were evaluated for the Danish population in a lifetime perspective. A discount rate of 3% per year was used for both costs and health outcomes.

For each of the included interventions costs and effects were plotted on the cost-effectiveness plane to illustrate cost-effectiveness, and cost-effectiveness ratios (CERs) were calculated compared to current practice. Current interventions primarily consisted of brief alcohol interventions conducted by GPs at an ad hoc basis, and not as part of an organised intervention. Thus, we were unable to create a comparator ‘null’ or ‘do nothing’ scenario due to heterogeneity in implementation and lack of evidence of effects and costs of current interventions. Cost-effectiveness ratios were calculated as the ratio of means[31].

Probabilities of the interventions being cost-effective are presented using cost-effectiveness acceptability curves. Since there is no agreed threshold of cost-effectiveness in Denmark, WHO's thresholds of less than the Gross Domestic Product (GDP) per capita for highly cost-effective and between one and three times GDP per capita for cost-effective were used as reference (GDP Denmark, 2009: € 39,900[32]). Using WHO's generalised cost-effectiveness approach[19], we also analysed the optimal sequence for implementing interventions: We analysed the cost-effectiveness of each intervention compared to a situation where none of the interventions were implemented, and ranked the interventions from most to least cost-effective. Subsequently, we evaluated the cost-effectiveness of intervention combinations. We estimated the total cost of the combined interventions as the sum of costs of the included interventions, whereas the total effect was estimated multiplicatively, since the percentage reduction obtainable by each additional intervention will only apply to the disease incidence that remains after previous interventions have been implemented. For each intervention in the optimal mix, we then calculated an incremental cost-effectiveness ratio (ICER), describing the cost-effectiveness of adding this intervention to the existing mix of interventions.

The statistical software SAS (version 9.2) was used for analyses of epidemiological data inputs and cost data. The cost-effectiveness analyses were performed in Excel (Microsoft Office 2007), applying the add-in programme Ersatz (version 1.31, Epigear 2012) for uncertainty analyses and calculation of combined intervention effects.

### Uncertainty and sensitivity analyses

We used Monte Carlo simulation methods to include uncertainty analyses into the cost-effectiveness model. Hereby the possible effects of uncertainty in estimates of relative risk, effects, costs and coverage rates of the intervention and cost offsets were assessed (for description of distributions used see appendix S1).

In sensitivity analyses we tested the effect of three main assumptions made in the modelling. The survey used for data on alcohol consumption only assessed current alcohol consumption in the Danish population. Former drinkers could therefore not be distinguished from lifetime abstainers. However, research has shown that drinkers might still be at higher risk of disease after becoming abstainers (i.e. former drinkers), compared to lifetime abstainers[33]. We tested the possible effect of not including former drinkers separately in our analysis by performing a maximum effect calculation: We estimated the intervention cost-effectiveness under the assumption that all people currently categorized as abstainers were former drinkers, applying estimates of relative risk for former drinkers to this group.

In the main analyses we modelled the cost-effectiveness per one year of intervention. This gives an indication of the cost and effects for every year an intervention is in place. In addition, we evaluated cost-effectiveness over a 10-year period, as suggested by WHO[19]. We used a decay rate of 2% for the taxation intervention (similar to the rate of inflation) and 50% for all other interventions. For the minimum legal drinking age intervention, the effects of 10 years of intervention was calculated by multiplying the one year effect by 10, accounting for an effect decay of 50% and a 3% discounting rate for costs and effects. This was done in order to capture the intervention effect on new 16-year olds being affected by the intervention, an effect that is not included in the model. This alternative decay assumption was more comparable to other cost-effectiveness studies of alcohol interventions[20], [21], [34].

Finally, we tested the implications of the disease trends integrated in the model (described in Appendix S2). In the sensitivity analysis we analysed intervention cost-effectiveness from a current level perspective, excluding the effects of trends in incidence and case fatality.

## Results

The cost of the interventions analysed in this study varied considerably: from € 0 for taxation to € 8.9 million for longer individual interventions (table 2). The effectiveness of the analyzed interventions, measured as DALYs averted per year of active intervention, also showed great variation: from 115 DALY for the long individual intervention to 2,853 DALY for a ban on alcohol advertisement. Generally interventions targeting the whole population (taxation, advertisement ban, regulation of opening hours) averted more DALY's than individual-focused interventions (brief and longer interventions).

**Table 2:** Cost-effectiveness of alcohol interventions for the Danish population aged 16+ (population in 2009: 4.5 million).

Intervention		DALYs prevented^a^			Cost offsets (€ million)			Intervention cost (€ million)			Net cost (€ million)			ICER^b^ (€/DALY)		
		Mean	CI95% low	CI95% high	Mean	CI95% low	CI95% high	Mean	CI95% low	CI95% high	Mean	CI95% low	CI95% high	Mean^c^	CI95% low	CI95% high
1.	30% taxation	1,911	1,524	2,292	−10.0	−13.2	−7.5	-	-	-	−10.0	−13.2	−7.5	Dominant	Dominant	Dominant
2.	Minimum legal drinking age	115	87	144	0.01	0.01	0.01	0.6	0.6	0.7	0.7	0.6	0.7	5,661	5,137	6,517
3.	Advertising bans	2,853	2,287	3,404	−17.2	−22.2	−13.0	0.4	0.3	0.4	−16.9	−21.8	−12.7	Dominant	Dominant	Dominant
4.	Reduced retail opening hours	2,163	1,760	2,569	−13.0	−16.5	−9.8	0.6	0.6	0.7	−12.3	−15.9	−9.1	Dominant	Dominant	Dominant
5.	Brief intervention	390	243	559	−2.4	−3.7	−1.5	2.2	1.8	2.7	−0.2	−1.5	0.9	Dominant	Dominant	1,579
6.	Longer intervention	129	82	188	−0.8	−1.2	−0.5	8.9	7.2	10.7	8.1	6.4	10.0	62,955	53,088	78,563

a DALY  =  disability-adjusted life year. ^b^ICER  =  incremental cost-effectiveness ratio. ^c^Calculated as ‘ratio of means’[31]

**Figure 1:**
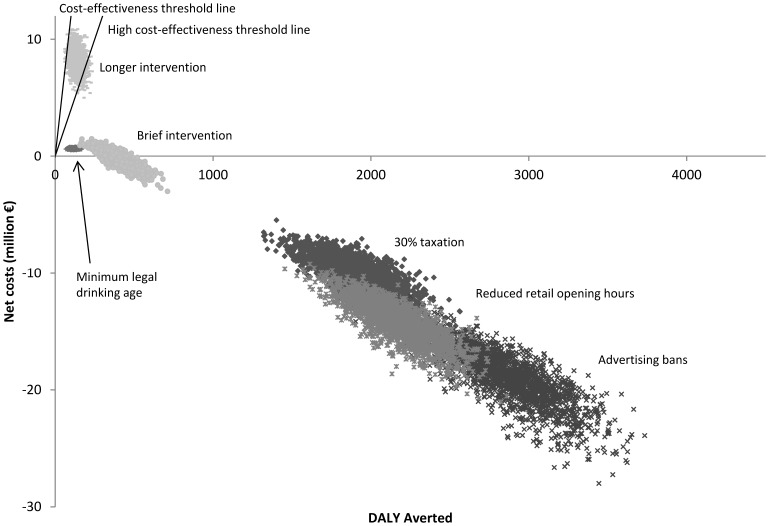
Cost-effectiveness of the six analysed interventions to reduce alcohol consumption (one year intervention time frame).

The advertisement ban, reduced opening hours and taxation are positioned in the south-east quadrant of the cost-effectiveness plane, indicating that these interventions are cost saving (figure 1). The brief intervention is positioned in both the north- and south-east quadrant, indicating that this intervention can be cost saving, but with a probability of less than one. Increased minimum legal drinking age and the longer individual alcohol intervention are positioned in the north-east quadrant. Minimum legal drinking age is below the line representing the threshold for ‘highly cost-effective’, whereas longer individual intervention is above this threshold, but below the ‘cost-effective’ threshold. Table 3 presents each intervention's probability of being cost saving and cost-effective.

**Table 3:** Probability of cost-effectiveness for alcohol interventions.

Intervention		Probability of being cost-saving	Probability of being very cost-effective (< € 40,000/DALY)	Probability of being cost-effective (< € 120,000/DALY)
1.	30% taxation	100%	100%	100%
2.	Minimum legal drinking age	0%	100%	100%
3.	Advertising bans	100%	100%	100%
4.	Reduced retail opening hours	100%	100%	100%
5.	Brief intervention	60%	100%	100%
6.	Longer intervention	0%	3%	99%

### Optimal combination of interventions

**Figure 2:**
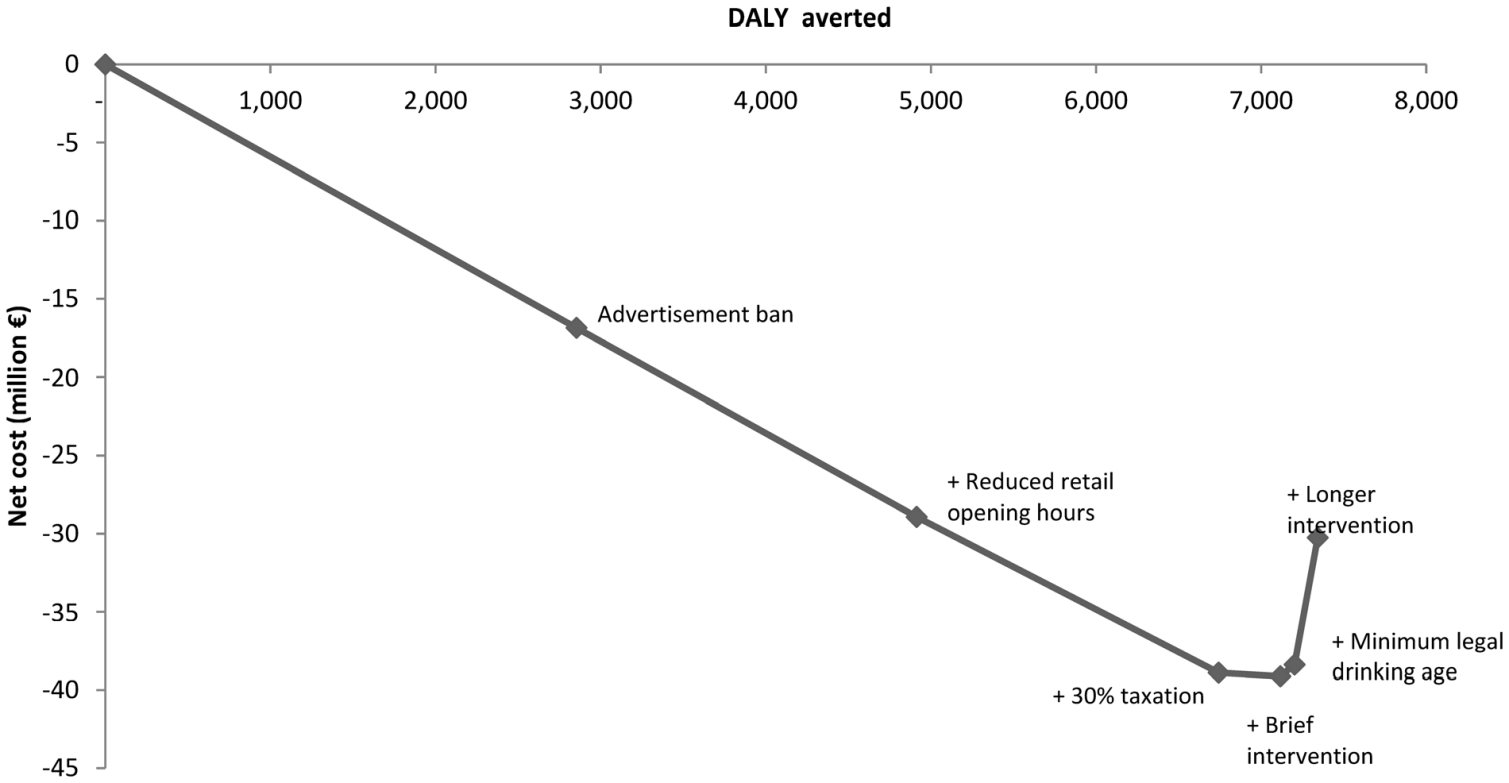
Optimal sequence for combining interventions to reduce alcohol consumption (one year intervention time frame).

The optimal order in which to implement the analyzed interventions is shown in figure 2. The optimal sequence for implementing interventions is entirely located in the cost-saving south-east quadrant of the cost-effectiveness plane, indicating that it could be cost saving to implement all six interventions. The advertisement ban at the beginning of the sequence achieves the largest gains in health and costs, followed by opening hours restrictions, taxation and brief individual interventions. Adding the minimum legal drinking age intervention or the longer individual intervention at the end of the sequence are not cost effective options. It should be noted that the order of the three interventions in the beginning of the sequence (advertisement ban, opening hours and taxation) is not certain since there is considerable overlap in the results of the uncertainty analysis of each interventions cost-effectiveness. The total effect of implementing all six interventions was 7,342 DALY and the net cost was € -30.2 million. If only the four cost-effective interventions were implemented the total effect would be 7,116 DALY. The cost of implementing these four interventions is € 3.3 million, which is compensated several times over by a total cost offset of € 42 million.

### Sensitivity analyses

Our sensitivity analyses showed that the assumption of identical risk profiles for former drinkers and lifetime abstainers only affected our results marginally, resulting in slightly higher cost-effectiveness ratios (see tables S1–S3).

Analysing the cost-effectiveness of the interventions applying the alternative time frame and decay rates, we still found the three legislative interventions targeting the whole population (taxation, advertisement ban and opening hours) to be cost saving. However, due to the differences in decay rates for each intervention, taxation was by far the most cost-effective intervention when analysed in the 10-year time frame. Minimum legal drinking age was still highly cost-effective. The individual interventions became less cost-effective, since the time frame increased whereas the decay rates were unchanged for these interventions and our model did not allow new cohorts to enter the model. The brief intervention was on the threshold of high cost-effectiveness, whereas the longer individual intervention was no longer cost-effective (see tables S1–S3).

When analysed without disease trends the interventions were generally more effective, since the decline in incidence seen over the past decades for most of the included diseases was no longer accounted for (see tables S1–S3). The effect was largest for the individually targeted interventions (brief and longer interventions), which were modelled with a decay of 50%. For the taxation intervention, the only intervention to affect all consumption levels, the effect was very small. Excluding trends from the analysis increased the incidence and case fatality of particularly cardiovascular diseases. Combined with an increased relative risk of cardiovascular diseases for low alcohol consumption compared with moderate consumption, this decreased the additional effect of the taxation intervention compared to the other interventions in the trend-free analysis. Cost offsets were smaller in the trend-free analysis (except for the minimum age intervention) compared to the main analysis. Cost offsets were affected by the changes in intervention effects and disease incidence and case fatality, but not by trends in health care expenditure since we were unable to include these in the main analysis.

## Discussion

We modelled the costs and effects of six interventions to prevent harmful alcohol consumption, and found that a ban on alcohol advertising, reduced hours of retail sales and increased taxation had the highest probability of being cost saving and should thus be first priority for investment. Our results are based on a model that simulates alcohol-related diseases and injuries in order to estimate the costs and health effects of implementing the alcohol interventions, and the optimal combination of these, in Denmark. The model draws on Danish patterns of alcohol consumption and local demographic and epidemiological data.

Some studies of cost-effectiveness of alcohol interventions have focussed on only one intervention, finding that taxation[17], price policies[18] and brief individual interventions[16] can be cost effective. However, comparability of cost-effectiveness between interventions is low when analysed in different studies. Few studies have evaluated the cost-effectiveness of several alcohol interventions implemented alone and in combination[20], [34]. For some interventions our results, show more favourable cost-effectiveness than found in these studies. In two linked studies Chisholm et al. analysed four of the interventions included in our study (taxation, advertisement bans, opening hours and brief interventions), and found that no interventions were cost saving for the subregion ‘Eur-A’, which includes Denmark[20], [21]. Chisholm et al. found taxation to be the most efficient intervention, with a favourable cost-effect ratio when evaluated against a per capita income threshold. For individual brief interventions we found a much lower effect compared to these studies, which is most likely due to Chisholm et al. assuming a higher coverage rate than we did [21].

We used an amended version of the model applied in the study by Cobiac et al.[34], which included five interventions comparable to the ones analysed in our study. This study found that in Australia taxation, bans on alcohol advertisements and an increased minimum legal drinking age were cost saving interventions. We did not find minimum legal drinking age to be cost-saving, which might be due to fewer prevented road traffic accidents in this age group, since the minimum legal age for driving is 18 in Denmark. However, we found the limited opening hours intervention to be cost saving, which was cost-effective but not cost-saving in the Australian study. When applying the alternative time frame and decay rates in our sensitivity analysis, we also find taxation to be the most cost-effective intervention.

The differences in intervention effects observed between our estimates and the results of other studies can partly be explained by differences in alcohol consumption and baseline epidemiology and demography. Further, some of the difference in cost-effectiveness is due to the interventions analysed in our study having low costs compared to scenarios analysed in other studies[20], [34]. For the legislative interventions (taxation, minimum legal drinking age, advertisement ban and limited hours of retail sale) estimates of intervention costs are based on the work done by a National Danish Prevention Taskforce, commissioned to examine and recommend preventive health interventions to be implemented in Denmark. Increased taxation was assumed to be cost neutral, since The Danish Ministry of Taxation estimated that current costs would not change with an increased taxation level[35]. This is not in accordance with WHO's generalised cost-effectiveness approach where interventions are compared to a null scenario[19]. However, even if taxation was assumed to have costs comparable to the other legislative interventions analysed, the intervention would still be cost-saving (results not shown). We also did not include changes in Government revenue in our cost estimates, since estimates of these were not available for all interventions. This is in line with the studies by Chisholm et al. and Cobiac et al.[20], [34]. Unlike in these studies, our estimates of intervention cost did not include patient-level costs. This might also explain some of the lower costs in our study, but only for the individually focused interventions which require attendance.

Seven thousand DALYs could be averted if the four cost-effective interventions in the optimal mix were implemented. This represents 19% of the reduction in DALY obtainable if the whole population consumed alcohol at a level below the lower recommended Danish threshold of one standard drink per day for women and two standard drinks per day for men[10]. The order of the three most cost-effective interventions in our optimal mix is not completely certain, since the estimates of costs and effects overlap (figure 2). Our results are thus comparable to the order found by Chisholm et al.[20], and by Cobiac et al.[34], and it is consistent with the conclusion by Chisholm et al. that population strategies (non-personal) are more cost-effective than personal strategies (targeting high risk persons)[21].

In the model we included trends in disease incidence and case fatality based on historical register data. Optimally we would have liked to also include trends in health care expenditure. However, we were unable to do so, since the diagnosis related groups (DRG) system, the main estimator of health care costs, is still evolving in Denmark and is not comparable across years. In the sensitivity analysis we tested the implications of including disease trends in the analyses. We found that intervention effects were generally larger and cost offsets smaller, leading to higher estimates of cost-effectiveness in the trend-free analysis.

We based the analyses of individually targeted interventions (brief and longer intervention) on estimates of costs and population coverage from an ongoing intervention in Copenhagen. However, estimates of effect are not yet available from this intervention, and we were unable to find estimates from other studies that exactly matched the type of brief and longer intervention. In the literature, we did not find evidence that longer interventions generally have added or more sustained effects compared to brief interventions[36]–[40]. We therefore used the same estimate of intervention effect for the two individually focused interventions. Since the repeated, longer intervention is more costly to implement than a brief telephone-based intervention, we naturally found brief interventions to be the more cost-effective of the two. However, the effect estimate used for both intervention types is based on studies analysing face-to-face interventions of between five and 30 minutes, sometimes with repeated intervention or booster sessions added[41]. This falls in between the interventions analysed in our study, which were a) a 15-minute telephone session and b) up to five one-hour face-to-face sessions. By using this estimate for both interventions in our analyses, we might therefore have overestimated the effect of brief interventions and underestimated the effect of longer interventions.

We were able to draw on the unique Danish population registers for information on disease incidence, mortality and costs for all individuals in the Danish population. Diseases are registered according to ICD-10 codes, whereas registration of injuries is based on the Nordic Classification of External Causes to Injuries[30]. A disadvantage of this approach is that long term disability due to injuries is not linked to injury incidence. Long term costs of injuries are thus not included in our cost estimates, which might therefore be underestimated[42].

For alcohol consumption we used data from a representative national Danish health survey[22], adjusted for underreporting. In our analyses we included average daily alcohol consumption and -for the injury calculations only- a rough estimate of frequency of binge drinking. Due to limitations in the available data, we were not able to properly include effects of drinking patterns. Differences have been found in the effect of alcohol intake among regular drinkers and irregular drinkers,[43] and this aspect should thus be investigated further in future studies.

The proportion of a tax increase that is passed on to consumers may be less than, equal to, or greater than the full change in taxation. Studies have shown pass-on rates for alcohol taxation of around 1.6–2.5, indicating that prices increase by more than the tax increase.[44], [45] Some differences were found between beverage types, and Kenkel also found that sales outlets with a higher baseline price passed less of the tax increase on to consumers.[44] Since baseline prices are rather high in Denmark, we expected a lower pass-on rate than found elsewhere, and we assumed a rather cautious pass-on rate of 1. However, if producers increase the price by more than the actual tax increase, the effect of alcohol taxation on alcohol consumption would be greater than found in our study.

Our analyses were based on the most recent data available. Some changes have, however, already occurred compared to the scenarios analyzed in our study. For example the level of taxation was increased in 2012 by 25% for beer and 55% for wine and then recently decreased by 15% for beer, and the minimum age for buying alcohol with an alcohol content of over 16.5% was raised to 18 years of age in 2011. Some of the potential health effects found in our study might therefore already have been obtained. However, the interventions implemented have not affected all types of alcohol evenly, and substitution might have reduced the health effects obtained.

A general limitation to cost-effectiveness analyses of alcohol interventions is that the evidence of effect on alcohol consumption for some of the interventions is relatively weak. This is especially true for advertising bans and control of hours of alcohol retail sale which have only been implemented and evaluated in a few places (corresponding to an evidence level of 2, according to the Oxford Levels of Evidence[46]). The cost-effectiveness results for these interventions should thus be interpreted with caution. Furthermore, little is known about the combined effect of multiple interventions. These shortcomings in the alcohol evidence base identify areas where further research on effects of alcohol interventions is needed.

In our modelling approach we did not include time lag effects in the temporal relationship between alcohol consumption and incidence of health outcomes. Few studies have examined this aspect, but a recent review found that there are immediate effects of changes in alcohol consumption on many health outcomes, except cancers, and that full effects are obtained after about 20 years[47].

Our model did not specifically account for higher health care costs in the last year of life. Studies have found that proximity to death is a better predictor for health expenditure than age and that models which do not include estimates of costs in last year of life separately overestimate the cost-effectiveness ratio[48]–[50]. However, as shown by Baal et al.[51], most of this effect is countered if the model includes costs of unrelated diseases, which were included in our model, and ICERs calculated accounting for unrelated diseases are found to be comparable to ICERs accounting for proximity to death.

In the cost-effectiveness modelling we used DALY as the measure of effect, a measure which has been widely discussed[52], [53]. We did not use age weighting, but both effects and costs were discounted by 3% and we assume unchanged disability weights. As argued by Chisholm et al., the main limiting factor related to the use of DALY in cost-effectiveness studies of alcohol is the inability to include non-health effects or effects on others than the person at risk[21]. An exception to this is victims of alcohol related accidents of crimes, but generally only direct effects on the person at risk are included.

This is in line with the health sector perspective applied in the analyses, where wider sickness or disability costs (e.g. lost productivity) were excluded. We were unable to access municipal data on social benefits, including sickness- and unemployment benefits. Since these aspects could not be included adequately, a health sector perspective was preferred. However, from a societal perspective, the interventions might be even more cost-effective than predicted in our study.

## Supporting Information

Appendix S1 **Intervention data sources and uncertainty assumptions.**
(DOCX)

Appendix S2 **Alcohol cost-effectiveness model.**
(DOCX)

Table S1 **Results of sensitivity analysis 1.**
(DOCX)

Table S2 **Results of sensitivity analysis 2.**
(DOCX)

Table S3 **Results of sensitivity analysis 3.**
(DOCX)
