# Supplementary material for: Cost-Effectiveness of Preventive Interventions to Reduce Alcohol Consumption in Denmark
Source: PLoS One. 2014 Feb 5;9(2):e88041. doi: 10.1371/journal.pone.0088041 (PMC3914889; doi:10.1371/journal.pone.0088041)
Supplement: Appendix S1 — Intervention data sources and uncertainty assumptions. (DOCX) [file pone.0088041.s001.docx]

Appendix S1

Intervention data sources and uncertainty assumptions

Input data to describe the six interventions analysed are taken from the epidemiological literature, as referenced in the table below, but also include preliminary estimates from interventions currently being implemented in Copenhagen, Denmark (unpublished), and results from the analyses done by a National Danish Prevention Taskforce (Forebyggelseskommisionen), set up to examine and recommend preventive health interventions to be implemented in Denmark[1].

The uncertainty in cost-effectiveness outputs is derived from uncertainty distributions around modelling inputs using Monte Carlo analysis. This involves repeated random sampling from the input parameter distributions, with evaluation of outputs at each iteration to determine a range of possible outcome values from which standard statistical parameters (e.g. mean, median) can be evaluated. We base our cost-effectiveness analysis of alcohol interventions on the outputs from 2,000 iterations, with input parameter distributions as follows:

| **Intervention** | | |
| --- | --- | --- |
| **Parameter** | **Value**  **Mean (SD)** | **Assumptions** |
| **1. 30% increased alcohol taxation**  Increased taxation of alcoholic beverages. In Denmark alcohol taxes vary according to beverage type, with heavier taxation on spirits than wine and beer. In the modelled scenario this is maintained, but with a 30% increase in taxation all types of alcoholic beverages. | | |
| Reduction in consumption | 2.1% (0.2) | Econometric modelling using price elasticity and cross-price elasticities[1].  Standard deviation assumed to be 10% of point estimate.  Distribution: Normal. |
| Proportion of population | 100% | This general legislative intervention is assumed to affect the entire population. |
| Total intervention cost | €0 | No extra costs assumed for administration, media and law enforcement compared to cost of current taxation[1]. |
| Baseline price and price after tax increase |  | Per litre pure alcohol: Beer €6.8, wine €6.7, spirits €20.1  Per litre pure alcohol: Beer €8.8, wine €8.7, spirits €26.1 |
| **2. Minimum legal drinking age of 18 years**  Increase in the minimum age for all purchasing or consumption of alcohol in public from 16 years to 18 years, through legislation and enforcement. | | |
| Reduction in consumption | Male: 32.8 g/day (3.3)  Female: 26.1 g/day (2.6) | Based on the effect of a change in the minimum legal drinking age from 15 to 16 years (changed in 2004)[1], the intervention is only assumed to affect harmful consumption. Average reduction is calculated as difference between hazardous and harmful consumption based on survey data[2]**.**  Standard deviation assumed to be 10% of point estimate.  Distribution: Normal. |
| Proportion of population | 12% (1.2) of 16-17 year old harmful drinkers. | This legislative intervention will only affect the population aged 16 and 17 years. The proportion of the population affected is based on the effect of a change in the minimum legal drinking age from 15 to 16 years[1].  Distribution: Beta. |
| Total intervention cost | Yearly: €375,000 (€37,500)  1^st^ year: €270,000 (€27,000) | Yearly costs include administration and increased enforcement of age limit[1].  One-time costs (1^st^ year) include media awareness campaign[1].  Standard deviation assumed to be 10% of point-estimate.  Distribution: Gamma. |
| **3. Advertising bans**  Comprehensive ban on all alcohol advertising via television, radio, billboards, etc. | | |
| Reduction in consumption | 4% (0.4%) | Range of 2% to 4% depending on alcohol prevalence, based on time series analyses[3]. 4% scenario modelled for Denmark due to high prevalence[1].  Standard deviation assumed to be 10% of point estimate.  Distribution: Normal. |
| Proportion of population | 100% of harmful drinkers | Intervention is expected to affect heavy alcohol used, defined in Chisholm et al. as an average consumption of more than 20g/day for women and 40g/day for men (equivalent to our consumption category ‘harmful’). |
| Total intervention cost | Yearly: €100,000 (€10,000)  1^st^ year: €270,000 (€27,000) | Yearly costs include administration and enforcement (based on current enforcement of tobacco advertisement-laws)[1].  One-time costs (1^st^ year) include media awareness campaign[1].  Standard deviation assumed to be 10% of point-estimate.  Distribution: Gamma. |
| **4. Reduced retail opening hours**  Restriction of purchase of alcohol, though legislation and enforcement, limiting the number of days of sale of alcohol (no retail sale on Saturdays). | | |
| Reduction in consumption | 3% (0.3) | Range of 1.5% to 3% depending on alcohol prevalence^2^. 3% scenario modelled for Denmark due to high prevalence[1,3].  Standard deviation assumed to be 10% of point estimate.  Distribution: Normal. |
| Proportion of population | 100% of harmful drinkers | Intervention is expected to affect heavy alcohol used, defined in Chisholm et al. as an average consumption of more than 20g/day for women and 40g/day for men (equivalent to our consumption category ‘harmful’). |
| Total intervention cost | Yearly: €375,000 (€37,500)  1^st^ year: €270,000 (€27,000) | Yearly costs include administration and increased enforcement[1].  One-time costs (1^st^ year) include media awareness campaign[1].  Standard deviation assumed to be 10% of point-estimate.  Distribution: Gamma. |
| **5. Brief individual intervention**  Intervention is open to all citizens, who can send a mobile phone text message to the consultation number, and subsequently receive a call from staff trained in motivational interviewing techniques. The duration of the alcohol consultation is typically 15 minutes. The brief intervention can be expanded by a longer intervention as described below (intervention 6.) | | |
| Reduction in consumption | 5.4 g/day (1.0) | Intention-to-treat estimate from a meta-analysis of randomised controlled trials[4].  Distribution: Normal. |
| Proportion of population | 3 % (0.3) of harmful drinkers | The project is currently being implemented in the municipality of Copenhagen. Preliminary estimates of coverage from this project are used (unpublished).  Standard deviation assumed to be 10% of point-estimate.  Distribution: Beta. |
| Intervention cost | Yearly: €2.2 m (€220,000) | Resources include mobile phone txt service and brief intervention conducted over the telephone by staff from the municipal preventive health centres trained in motivational counselling (based on unpublished estimated costs from the municipality of Copenhagen).  Standard deviation assumed to be 10% of point-estimate.  Distribution: Gamma. |
| **6. Longer individual intervention**  Face to face alcohol consultation conducted in municipal prevention centres by staff trained in motivational interviewing. People with harmful alcohol consumption are offered 1 to 5 consultations of approximately 1 hour duration. | | |
| Reduction in consumption | 5.4 g/day (1.0) | Intention-to-treat estimate from a meta-analysis of randomised controlled trials[4].  Distribution: Normal. |
| Proportion of population | 1% (0.1) of harmful drinkers | The project is currently being implemented in the municipality of Copenhagen. Preliminary estimates of coverage from this project are used (unpublished).  Standard deviation assumed to be 10% of point-estimate.  Distribution: Beta. |
| Total intervention cost | Yearly: €8.9 m (€890,000) | Resources include up to five intervention sessions of about one hour with trained staff from the municipal preventive health centre.  Resources include mobile phone txt service and brief intervention conducted over the telephone by staff from the municipal preventive health centres trained in motivational counselling (based on unpublished estimated costs from the municipality of Copenhagen).  Standard deviation assumed to be 10% of point-estimate.  Distribution: Gamma. |
| **All interventions** | | |
| Relative risks (RRs) of disease | Table 3  (supplement 1) | See Table 3 in supplement 2.  Distribution: Normal (ln RR). |
| Disease and injury cost offsets | Table 4  (supplement 1) | Calculated based on Danish cost data (see supplement 2 for details on calculation methods).  Distribution for costs: Gamma  Standard deviation assumed to be 10% of point-estimate.  Distribution for cost offsets: Normal. |
| NB. All costs have been converted to Euro (€1 = 7.45 DKK (May 13 2013)) | | |

Reference List

1. Forebyggelseskommisionen (2009) Vi kan leve længere og sundere - Forebyggelseskommisionens anbefalinger til en styrket forebyggende indsats [We can live longer and healthier - The Prevention Commission's recommendations for strengthened prevention].

2. Danish Health and Medicines Authority (2011) Den Nationale Sundhedprofil 2010 - Hvordan har du det? [The National Health Profile 2010 - How are you doing?].

3. Chisholm D, Rehm J, Van OM, Monteiro M (2004) Reducing the global burden of hazardous alcohol use: a comparative cost-effectiveness analysis. J Stud Alcohol 65: 782-793.

4. Bertholet N (2005) Reduction of alcohol consumption by brief alcohol intervention in primary care: Systematic review and meta-analysis. Archives of Internal Medicine 165: 986-995. doi: 10.1001/archinte.165.9.986.
