# Supplementary material for: Cost-Effectiveness of Preventive Interventions to Reduce Alcohol Consumption in Denmark
Source: PLoS One. 2014 Feb 5;9(2):e88041. doi: 10.1371/journal.pone.0088041 (PMC3914889; doi:10.1371/journal.pone.0088041)
Supplement: Appendix S2 — Alcohol cost-effectiveness model. (DOCX) [file pone.0088041.s002.docx]

Appendix S2

Alcohol cost-effectiveness model

The work reported in this paper builds on the methods developed in the Australian study ‘Assessing Cost-Effectiveness in Prevention’ (ACE-Prevention)[1], with a focus on the work done regarding alcohol (ACE Alcohol)[2]. For the purpose of the current study, the model has been fitted with Danish data, selected assumptions have been changed, and the model has been improved based on the previous experiences.

This supplement file describes the model, the input data used and the assumptions made in the modelling (for a comparison with the original Australian model see Cobiac et al., 2009[2]).

**1. Cost-effectiveness model for alcohol interventions**

The modelling approach used evaluates cost-effectiveness of alcohol interventions over the lifetime of the Danish population in 2009. Data sources used include: intervention costs and effects from the alcohol intervention literature, work done by “Forebyggelseskommisionen”, a Danish Governmental Commission commissioned to examine and recommend preventive health interventions to be implemented in Denmark, and Danish register data on disease epidemiology and costs of disease treatment. The model runs in Excel (Microsoft Office 2007) using the add-in tool Ersatz (Version 1.31, Epigear 2012) for uncertainty analysis.

The model simulates the impact of eight different alcohol interventions on population health (see supplement file 1 for a description of the modelled interventions). The aim of the interventions studied is to reduce risk of harm by reducing alcohol consumption to low risk levels. These changes in alcohol consumption affect the mortality and prevalence of alcohol-related diseases, and mortality and incidence of alcohol-related injuries, which influence overall rates of mortality and disability in the population. In the model, health effects are measured in disability-adjusted life years (DALYs) averted.

DALYs averted by alcohol interventions are estimated as the difference in health-adjusted years of life lived between a Danish population that continues to drink alcohol at current rates and an identical population that receives the intervention and reduces alcohol consumption. The health-adjusted years of life lived by each of the two populations are calculated by dividing the populations into age cohorts (16-17 year olds, 18-19 year olds and five-year age groups from age 20-24 to age 95+), and simulating each cohort in a multi-state life table until everyone has either died or reached 100 years of age. Years of life lived are adjusted at each age for time spent in poor health due to disease or injury.

For the calculation of difference in DALYs we used estimates of overall prevalent YLD^[[1]](#footnote-1)^ from the Australian Burden of Disease study 2003, since no estimates of pYLD currently exist for Denmark^[[2]](#footnote-2)^.

Based on the available evidence, the interventions are modelled with two different time frames for sustainability of intervention effect. Some evidence exists for the sustainability of the effects of Brief Alcohol Interventions aimed at individuals[4]. The effects of these interventions on alcohol consumption are therefore modelled assuming a 50% decay rate after the first year, implying that the intervention effect will last for about 10 years. However, for most population based alcohol interventions there is a lack of evidence on future sustainability of effect. We modelled these interventions with a decay rate of 100%, implying that the intervention effects will be sustained only for the year in which the intervention is implemented and enforced. The main reason for this very high decay rate was to increase comparability of the cost-effectiveness results, since some interventions affect the whole population, whereas the intervention of increased minimum legal drinking age affects only an age-specific subgroup of the population. Thus, by modelling the cost-effectiveness per year of intervention, we obtain more comparable results across the included interventions.

This choice of decay rate is different from the approach taken in the Australian ACE-Alcohol study[2] and is also not in accordance with WHO, who have suggested to evaluate intervention effects over a 10-year period[5]. However, this approach assumes that interventions that target individuals are repeated on an annual basis to a new portion of the population. To test the implications of the decay rates applied in our model, the decay rates used in the Australian study are applied in a sensitivity analysis.

**2. Alcohol consumption**

The risk factor alcohol consumption is divided into four levels – abstinence, low, hazardous and harmful (Table 1) – based on the Danish Health and Medicines Authority recommendations for alcohol consumption[6]. The categorisation is based on number of standard drinks consumed per week, recalculated into grams per day (1 standard drink = 12 grams of alcohol[6]). Data collected in The National Health Profile 2010 for Denmark are used to determine the average consumption of alcohol at each level in Denmark[7].

Studies have shown that population surveys tend to underestimate the actual adult per capita alcohol consumption[8]. The estimate of alcohol consumption from survey data is therefore adjusted for underreporting, using the coverage rate between survey data and adult per capita alcohol consumption from sales statistics (as described by Rehm et al.[9]). To avoid over-adjustment, we assume that 10% of adult per capita consumption estimated from sales statistics is not consumed (as suggested by Taylor et al.[10]). The alcohol consumption level registered in The Danish National Health Profile 2010 accounted for 72% of this 90%-level from sales statistics.

This adjustment approach presumes that under-coverage by surveys is evenly distributed in the population, which might not be the case. However, there is no clear evidence on differential underreporting of alcohol consumption by different subpopulations in surveys[9]. Table 2 shows the adjusted average consumption of alcohol at each consumption level. Prevalence of the four levels of consumption was calculated for each age group in the model, and is shown in Figure 1 for the entire Danish population.

Table 1. Definition of Danish categories of alcohol consumption (grams per day)[6].

| Category | Males | Females |
| --- | --- | --- |
| Abstinence | <1.7 | <1.7 |
| Low | 1.7-23.9 | 1.7-11.9 |
| Hazardous | 24-35.9 | 12-23.9 |
| Harmful | >36 | >24 |

Table 2. Average alcohol consumption within consumption categories (grams per day)[7].

| Category | Males | Females |
| --- | --- | --- |
| Abstinence | 0.0 | 0.0 |
| Low | 12.0 | 6.3 |
| Hazardous | 31.3 | 18.3 |
| Harmful | 73.3 | 43.8 |

Figure 1. Distribution of the Danish population on alcohol consumption categories[7]

3. Modelling health outcomes

The model simulates intervention effects on health outcomes associated with alcohol consumption. Based on epidemiological evidence the following disease outcomes are included in the model: Ischaemic heart disease, ischaemic and hemorrhagic stroke, hypertensive heart disease, pancreatitis, cirrhosis, cancer of the breast (in women), mouth and oropharynx, oesophagus, liver, larynx, colon and rectum and alcohol dependence[11–17]. Further, excess consumption of alcohol increases the risk of a wide range of injury outcomes due to road traffic accidents (RTA) and other accidents (non-RTA)[10,18,19]. Non-RTA injuries associated with death or disability due to alcohol consumption include falls, fires, burns and scalds, drowning, other accidents, suicide and self-inflicted injuries, and homicide and violence[20].

3.1 Disease outcomes (incl. alcohol dependence)

Each of the alcohol-related diseases is modelled using a set of differential equations that describe the transitions of people between four states (healthy, diseased, dead from the disease, and dead from all other causes). Transition of people between the four states is based on rates of mortality, incidence, case fatality and remission[21]. These epidemiological data inputs are obtained from Danish register data from Statistics Denmark, with additional use of DISMOD II[21] to enforce internal consistency of the data and derive parameters not available from the registers.

For ischaemic heart disease, stroke, hypertensive heart disease, cirrhosis and all cancers, case fatality and prevalence are estimated using DISMOD II, based on incidence and mortality data from The National Patient Register (NPR)[22] and The Register of Causes of Death[23], with remission set to zero in DISMOD II. Incidence estimates included only those who had not been diagnosed with the same condition in the five years (2003-2008) prior to the year of study. Due to a shorter duration of the disease, pancreatitis is modelled based on mortality[23] and YLD[24]. For alcohol dependence, incidence and case fatality are estimated using DISMOD II, based on prevalence of alcohol dependence (calculated from Hvidtfeldt et al.[25,26]), rate of remission[27] and relative risk of mortality[unpublished data from Global Burden of Disease 2010, personal communication with Theo Vos] as input.

Average disability associated with each disease (disability weights) was derived from Australian Burden of Disease calculations[28], since such calculations are not available for Denmark. These disability weights are a combination of disability weights from the original GBD study[29], the disability weights used in Dutch Burden of Disease study[30] and few weights derived locally for Australia[28].

Adjustment for future changes in disease incidence and case fatality is based on trend analysis of mortality and incidence rates by cause. This is the same –slightly simplified– approach as in the Australian ACE-alcohol study[2]. Mortality trends are based on mortality between 1977 and 2009 for cardiovascular diseases and breast cancer, and between 1994 and 2009 for other cancers and cirrhosis, due to lack of available comparable data. Incidence trends are based on data from 1977 to 2009, but only available for cancers[31]. For pancreatitis and alcohol dependence we have assumed that rates are stable over time.

Mortality trends for cirrhosis were assumed to be fully due to changes in incidence, and the incidence trend was therefore adjusted to reflect changes in mortality over the projection period, with case-fatality held constant. For cardiovascular diseases, findings from Unal et al. suggest that 58% of the drop in mortality from coronary heart disease observed in England and Wales between 1981 and 2000 was due to a drop in incidence and the remaining 42% due to a reduction in case-fatality[32]. The same proportions were assumed to apply in our model to all cardiovascular disease over the projection period. For cancers, changes in case fatality were estimated based on mortality and incidence trends. Past trends were assumed to continue over the next 20 years with disease rates to remain constant thereafter.

3.1.1 Intervention effects on disease outcomes

For all disease outcomes except alcohol dependence the intervention effect on disease incidence is modelled by a modified version of the potential impact fraction[33,34]. In this version the intervention effect changes the relative risk of disease in the intervention population rather than the population prevalence of alcohol consumption (Equation 1). This modification allows interventions to be modelled from their impact on alcohol consumption (i.e. in standard units, serves or grams per day), a continuous measure of effect, rather than modelled from their change in population prevalence of each consumption category, which results in non-linear effects from regular increases in effect size.

(1)

∑

∑

∑

=

=

=

−

=

*n*

*i*

*i*

*i*

*n*

*i*

*i*

*i*

*n*

*i*

*i*

*i*

1

1

1

RR

p

RR'

p

RR

p

PIF

where:

PIF is the potential impact fraction;

p*_i_* is the prevalence of alcohol consumption at exposure level *i*;

RR*_i_* is the relative risk of disease associated with alcohol consumption at exposure level *i*; and

RR'*_i_* is the relative risk of disease associated with alcohol consumption after an intervention is implemented in the population at exposure level *i*.

The relative risks applied in the model are derived from existing meta-analyses of data describing the relationship between alcohol consumption and the risk of alcohol related outcomes (see table 3). Estimates of relative risk at baseline and after intervention are calculated for average alcohol consumption within each consumption category (RR and RR’ in equation 1) based on relative risk functions and covariance matrices for each association between alcohol consumption and the included health outcomes (see Table 3 for baseline estimates and references).

For alcohol dependence, which is wholly attributable to excess alcohol consumption, the intervention effect on disease incidence is modelled, by age and sex, as a reduction in the incidence of alcohol dependence that is proportional to the change in consumption at a harmful level of alcohol consumption (Equation 2). Incidence of alcohol dependence is assumed to be negligible below a harmful level of consumption.

(2)

⎟

⎟

⎠

⎞

⎜

⎜

⎝

⎛

−

Δ

−

=

′

*Abs*

*Harm*

*Harm*

*i*

*i*

c

c

c

1

where:

*i’* is the incidence of alcohol dependence after an intervention is implemented in the population;

*i* is the current incidence of alcohol dependence, adjusted for level of dependence among harmful drinkers;

Δc*_Harm_* is the average change in alcohol consumption in the population due to an intervention, in g/day, among those currently drinking at a harmful level;

c*_Harm_* is the average alcohol consumption, in g/day, among those currently drinking at a harmful level; and

c*_Abs_* is the average alcohol consumption, in g/day, among those currently defined as abstinent.

3.2 Injury outcomes

Injuries are acute in nature and changes in injury outcomes due to alcohol interventions are therefore modelled through direct changes in injury-related mortality and disability. Mortality inputs for injuries are derived from The Danish Injury Register[35]. For disability, injury YLD rates for RTA and non-RTA respectively were derived from WHO’s estimates for the sub region EUR-A (Western Europe), since such calculations are not available for Denmark.

Estimates of incidence of injuries, for the cost calculations (see section 4), are derived from Statistics Denmark based on data from The National Patient Register (NPR)[22]. Registration of injuries is based on the Nordic Classification of External Causes to Injuries. After the first contact, treatment of injury effects are normally not linked to the injury event and long term disability due to injuries are thus not included in our estimates.

As for the included diseases, future changes in incidence and mortality of injuries are estimated from trend analysis of mortality and injury rates by cause. However, due to lack of historical data on non-RTA injuries and incidence of RTA injuries, we are only able to model mortality trend for RTA injuries. These are based on mortality between 1977 and 2009. Incidence trends were assumed to reflect changes in mortality over the projection period, assuming unchanged case-fatality. Past trends were assumed to continue over the next 20 years with injury rates to remain constant thereafter.

3.2.1 Intervention effects on injury outcomes

The effects of alcohol interventions on injuries are measured by translating a change in alcohol consumption into a change in mortality and morbidity from injuries, by calculating Alcohol-Attributable Fractions (AAF), an adaptation of the potential impact fraction (PIF), as outlined by Taylor et al.[10].

The injury morbidity AAF is calculated by Equation 3, based on time at risk of injuries (calculated from alcohol metabolism rates) and two dimension of alcohol consumption: binge drinking (here defined as weekly drinking more than five standard drinks in one occasion) and alcohol consumption on non-binge days. However, for RTA, the AAF for women was calculated by multiplying the AAF for men by the mean consumption of alcohol for women divided by the mean consumption of alcohol for men[36].

$AAF=\frac{(P_{abs}RR_{abs}+\sum_{i=1}^{n} P_{non-binge(i)}RR_{non-binge(i)}+\sum_{i=1}^{n} P_{binge(i)}RR_{binge(i)})-(P_{abs}RR_{abs}+\sum_{i=1}^{n} P_{non-binge(i)}RR'_{non-binge(i)}+\sum_{i=1}^{n} P_{binge(i)}RR'_{binge(i)})}{(P_{abs}RR_{abs}+\sum_{i=1}^{n} P_{non-binge(i)}RR_{non-binge(i)}+\sum_{i=1}^{n} P_{binge(i)}RR_{binge(i)})}$ (3)

where:

P_abs_ and RR_abs_ is the prevalence and relative risk for current abstainers

P_binge(i)_ and RR_binge(i)_ is the prevalence and relative risk for current drinkers who engage in binge drinking, for alcohol consumption at exposure level *i*

P_non-binge(i)_ and RR_non-binge(i)_ is the prevalence and relative risk for current drinkers who do not engage in binge drinking, for alcohol consumption at exposure level *i*

RR'_binge/non-binge(i)_ is the relative risk of injury associated with alcohol consumption at exposure level *i* after an intervention is implemented, for current drinkers who do/do not engage in binge drinking

with consumption level *i* being low, hazardous or harmful as outlined in section 2 (Table 1).

To estimate the injury mortality AAF from road traffic accidents, we multiply the morbidity AAF for RTA by 3/2. Similarly, to estimate the mortality AAF due to non-road traffic accidents, we multiply the morbidity AAF for non-RTA by 9/4[36]. These multipliers are based on two studies comparing blood alcohol levels of emergency room patients with blood alcohol levels of patients who died from injuries, obtained from coroners’ reports[37,38].

4. Disease and injury costs

Costs of prevented health care use (cost offsets), due to reduced rates of alcohol-related disease and injury, are analyzed from a health sector perspective. Cost offsets include costs of treatment, health care personnel, medication etc., but exclude costs such as costs to the individual or caregivers, costs due to lost productivity or costs associated with alcohol-related crime and violence.

Costs are evaluated using data on costs of treatment in the health care system, including inpatient and out-patient costs (from the Diagnosis Related Grouping (DRG) Register[35]), data on costs related to consultations with general practitioners and specialists (from the Danish National Health Service Register[39]), data on costs of pharmaceuticals (from the Danish National Prescription Register[40]) and data on municipal health care costs, such as nursing homes, home nursing, rehabilitation etc. Health care costs borne by municipalities are generally not consistently reported across municipalities in Denmark. We therefore assessed municipal health care costs in the municipality of Copenhagen^^[[3]](#footnote-3)^^ (based on data from the Health and Care Administration of the City of Copenhagen[Unpublished data], linked to register data on health outcomes via Statistics Denmark).

In the absence of a Danish cost of illness study, cost offsets are quantified for each of the included health outcomes by comparing health care costs for people with the disease and health care costs for people without the disease. Multiple regression analyses were used, mutually controlling for all included health outcomes and for other diseases not associated with alcohol consumption. Estimates were obtained by sex and age (<49, 50-69, 70+ years), based on rates of disease in 2009 (Table 4). Due to the regression approach to cost estimation, negative costs could occur for outcomes that mainly affect otherwise healthy people, such as accidents. In our analyses we found negative costs for RTA for women aged <50 and for non-RTA for both men and women aged <50. All three of these costs estimates were relatively small (below €450 per incident case), and in the model these negative costs were set to €1. This approach to cost estimation might have caused an underestimation of the true cost-effectiveness of the interventions, especially for the minimum legal drinking age intervention.

Costs are assumed to accrue per prevalent case for the cardiovascular diseases, cirrhosis and alcohol dependence, and per incident case for pancreatitis, injuries and all cancers, which often have a shorter duration of illness (Table 4). Estimates of cost per incident case were based on costs for only those who had not been diagnosed with the given condition five years (2003-2008) prior to the year of study - except for injuries.

Average health care costs (by age and sex) due to diseases not associated with alcohol consumption were included in the analysis in order to account for costs in added years of life.

Table 3. Baseline relative risks of disease due to alcohol consumption

|  |  | Alcohol intake level | | | |  |
| --- | --- | --- | --- | --- | --- | --- |
| Disease | Sex | Abstinence | Low | Hazardous | Harmful | Source |
| Ischaemic heart disease (15-34yr) | Male  Female | 0.95 (0.83-1.08)  0.93 (0.89-0.97) | 0.64 (0.17-1.47)  0.34 (0.12-0.67) | 0.56 (0.17-1.19)  0.34 (0.06-0.87) | 1.00  1.01 (0.05-4.69) | Roerecke and Rehm, 2011[41] |
| Ischaemic heart disease (35-64yr) | Male  Female | 0.97 (0.90-1.04)  0.96 (0.94-0.98) | 0.75 (0.38-1.23)  0.53 (0.31-0.77) | 0.70 (0.37-1.10)  0.51 (0.21-0.90) | 1.00  0.87 (0.20-2.36) | Roerecke and Rehm, 2011[41] |
| Ischaemic heart disease (65+yr) | Male  Female | 1.00 (0.96-1.03)  1.00 (0.97-1.03) | 0.99 (0.64-1.39)  0.99 (0.54-1.66) | 0.98 (0.62-1.42)  1.00 (0.46-1.86) | 1.00  1.02 (0.50-1.75) | Roerecke and Rehm, 2011[41] |
| Ischaemic stroke | Male  Female | 1.00  1.00 | 0.87 (0.81-0.93)  0.84 (0.76-0.91) | 0.97 (0.90-1.04)  0.84 (0.74-0.94) | 1.24 (1.12-1.37)  0.98 (0.86-1.12) | Patra et al., 2010[12] |
| Hemorrhagic stroke | Male  Female | 1.00  1.00 | 1.10 (1.06-1.14)  0.66 (0.52-0.83) | 1.27 (1.15-1.40)  0.76 (0.57-0.99) | 1.77 (1.40-2.20)  1.13 (0.81-1.54) | Patra et al., 2010[12] |
| Hypertensive heart disease | Male  Female | 1.00  1.00 | 1.12 (1.09-1.14)  0.80 (0.69-0.92) | 1.33 (1.25-1.41)  1.15 (0.89-1.45) | 1.95 (1.69-2.24)  2.39 (1.61-3.42) | Taylor et al., 2009[11] |
| Pancreatitis | Male  Female | 1.00  1.00 | 1.02 (1.02-1.03)  1.01 (1.01-1.01) | 1.16 (1.12-1.20)  1.05 (1.04-1.07) | 2.26 (1.88-2.69)  1.34 (1.25-1.34) | Irving et al., 2009[13] |
| Cirrhosis | Male  Female | 1.00  1.00 | 1.23 (1.17-1.28)  1.82 (1.63-2.04) | 1.70 (1.51-1.90)  2.76 (2.27-3.32) | 3.49 (2.63-4.53)  4.81 (3.55-6.35) | Rehm et al., 2010[14] |
| Breast cancer | Male  Female | –  1.00 | –  1.06 (1.05-1.07) | –  1.17 (1.14-1.21) | –  1.47 (1.38-1.57) | Corrao et al., 2004[16] |
| Mouth and oropharynx cancer | Male  Female | 1.00  1.00 | 1.37 (1.33-1.41)  1.18 (1.16-1.20) | 2.13 (2.00-2.27)  1.59 (1.53-1.66) | 4.58 (4.13-5-06)  2.77 (2.55-2.99) | Corrao et al., 2004[16] |
| Oesophagus cancer | Male  Female | 1.00  1.00 | 1.17 (1.16-1.18)  1.09 (1.08-1.09) | 1.51 (1.47-1.54)  1.27 (1.26.1.29) | 2.59 (2.45-2.74)  1.78 (1.72-1.84) | Corrao et al., 2004[16] |
| Liver cancer | Male  Female | 1.00  1.00 | 1.09 (1.06-1.12)  1.05 (1.03-1.06) | 1-24 (1.15-1-34)  1.14 (1.09-1.19) | 1.59 (1.36-1.85)  1.35 (1.22-1.49) | Corrao et al., 2004[16] |
| Larynx cancer | Male  Female | 1.00  1.00 | 1.19 (1.17-1.21)  1.09 (1.08-1.10) | 1.55 (1.49-1.62)  1.30 (1.26-1.33) | 2.76 (2.50-3.03)  1.85 (1.75-1.97) | Corrao et al., 2004[16] |
| Colon cancer | Male  Female | 1.00  1.00 | 1.02 (1.01-1.04)  1.01 (1.00-1.02) | 1.06 (1.02-1.11)  1.04 (1.01-1.06) | 1.15 (1.04-1.27)  1.09 (1.03-1.15) | Corrao et al., 2004[16] |
| Rectal cancer | Male  Female | 1.00  1.00 | 1.04 (1.03-1.05)  1.02 (1.02-1.03) | 1.12 (1.09-1.15)  1.07 (2.05-1.08) | 1.29 (1.21-1.38)  1.17 (1.12-1.21) | Corrao et al., 2004[16] |
| NB. Values are mean relative risk and 95% confidence interval at average alcohol consumption for each intake category (Table 2), calculated by Monte Carlo analysis with 3000 iterations. | | | | | | |

Table 4. Annual cost per prevalent or incident case of disease

| **Disease** | **Units** | **Sex** | **Cost (€)** | | |
| --- | --- | --- | --- | --- | --- |
|  |  |  | < 50 years | 50-69 years | 70+ years |
| Ischaemic heart disease | €/prevalent case | Men | 8,947 | 10,001 | 10,286 |
|  |  | Women | 7,904 | 7,770 | 9,164 |
| Ischaemic stroke | €/prevalent case | Men | 15,082 | 14,936 | 15,352 |
|  |  | Women | 14,496 | 14,886 | 15,372 |
| Hemorrhargic stroke | €/prevalent case | Men | 24,525 | 24,519 | 22,134 |
|  |  | Women | 26,426 | 30,003 | 20,335 |
| Hypertensive heart disease | €/prevalent case | Men | 10,709 | 8,201 | 12,442 |
|  |  | Women | 5,016 | 7,970 | 9,874 |
| Pancreatitis | €/prevalent case | Men | 9,458 | 10,385 | 9,596 |
|  |  | Women | 11,498 | 11,474 | 9,158 |
| Cirrhosis | €/prevalent case | Men | 13,526 | 10,825 | 9,985 |
|  |  | Women | 8,845 | 7,911 | 6,472 |
| Breast cancer | €/prevalent case | Men | - | - | - |
|  |  | Women | 10,384 | 9,876 | 8,707 |
| Mouth & oropharynx cancer | €/incident case | Men | 12,481 | 14,763 | 8,521 |
|  |  | Women | 11,382 | 12,127 | 11,226 |
| Oesophageal cancer | €/incident case | Men | 22,882 | 23,638 | 19,083 |
|  |  | Women | 43,745 | 26,606 | 16,639 |
| Liver cancer | €/incident case | Men | 18,705 | 16,873 | 16,495 |
|  |  | Women | 24,006 | 22,762 | 12,385 |
| Larynx cancer | €/incident case | Men | 10,636 | 8,127 | 11,828 |
|  |  | Women | 7,049 | 5,746 | 7,869 |
| Colon cancer | €/incident case | Men | 18,711 | 17,232 | 17,167 |
|  |  | Women | 17,775 | 17,028 | 17,031 |
| Rectal cancer | €/incident case | Men | 18,288 | 15,595 | 13,427 |
|  |  | Women | 13,066 | 16,609 | 14,877 |
| Road traffic accidents | €/incident case | Men | 617 | 717 | 1,977 |
|  |  | Women | 1^a^ | 140 | 285 |
| Non-road traffic accidents | €/incident case | Men | 1^a^ | 145 | 2,717 |
|  |  | Women | 1^a^ | 404 | 3,060 |
| Alcohol dependence | €/incident case | Men | 3,675 | 4,417 | 4,550 |
|  |  | Women | 3,934 | 4,344 | 4,610 |
| Other disease | Average €/person in the population | Men | 444 | 780 | 1,123 |
|  |  | Women | 777 | 755 | 1,207 |
| ^a^ Negative costs were set to €1. See section 4. “Disease and injury costs” above. | | | | | |

Reference List

1. Vos T, Carter R, Barendregt JJ, Mihalopoulos C, Veerman JL, et al. (2010) Assessing Cost-Effectiveness in Prevention (ACE-Prevention): Final Report.

2. Cobiac L, Vos T, Doran C, Wallace A (2009) Cost-effectiveness of interventions to prevent alcohol-related disease and injury in Australia. Addiction 104: 1646-1655. 10.1111/j.1360-0443.2009.02708.x [doi].

3. WHO (2009) Metrics: Disability-Adjusted Life Year (DALY). who int/healthinfo/global_burden_disease/metrics_daly/en/index html .

4. Nilssen O (2004) Long-term effect of brief intervention in at-risk alcohol drinkers: a 9-year follow-up study. Alcohol Alcohol 39: 548-551. 10.1093/alcalc/agh095 [doi];agh095 [pii].

5. World Health Organization (2003) Making choices in health: WHO guide to cost-effectiveness analysis.

6. Danish Health and Medicines Authority (2012) Udmeldinger om Alkohol [Recommendations regarding alcohol consumption]. <http://www> sst dk/Sundhed%20og%20forebyggelse/Alkohol/UdmeldingerOmAlkohol aspx .

7. Danish Health and Medicines Authority (2011) Den Nationale Sundhedprofil 2010 - Hvordan har du det? [The National Health Profile 2010 - How are you doing?].

8. Rehm J, Klotsche J, Patra J (2007) Comparative quantification of alcohol exposure as risk factor for global burden of disease. Int J Methods Psychiatr Res 16: 66-76. 10.1002/mpr.204.

9. Rehm J, Kehoe T, Gmel G, Stinson F, Grant B (2010) Statistical modeling of volume of alcohol exposure for epidemiological studies of population health: the US example. Popul Health Metr 8: 3. 1478-7954-8-3 [pii];10.1186/1478-7954-8-3 [doi].

10. Taylor B, Shield K, Rehm J (2011) Combining best evidence: A novel method to calculate the alcohol-attributable fraction and its variance for injury mortality. BMC Public Health 11: 265. 10.1186/1471-2458-11-265.

11. Taylor B, Irving HM, Baliunas D, Roerecke M, Patra J, et al. (2009) Alcohol and hypertension: gender differences in dose−response relationships determined through systematic review and meta-analysis. Addiction 104: 1981-1990. 10.1111/j.1360-0443.2009.02694.x.

12. Patra J, Taylor B, Irving H, Roerecke M, Baliunas D, et al. (2010) Alcohol consumption and the risk of morbidity and mortality for different stroke types--a systematic review and meta-analysis. BMC Public Health 10: 258. 1471-2458-10-258 [pii];10.1186/1471-2458-10-258 [doi].

13. Irving HM, Samokhvalov AV, Rehm J (2009) Alcohol as a risk factor for pancreatitis. A systematic review and meta-analysis. JOP 10: 387-392. v10i04a09 [pii].

14. Rehm J, Taylor B, Mohapatra S, Irving H, Baliunas D, et al. (2010) Alcohol as a risk factor for liver cirrhosis: a systematic review and meta-analysis. Drug Alcohol Rev 29: 437-445. DAR153 [pii];10.1111/j.1465-3362.2009.00153.x [doi].

15. Baan R, Straif K, Grosse Y, Secretan B, El Ghissassi F, et al. (2007) Carcinogenicity of alcoholic beverages. The Lancet Oncology 8: 292-293. doi: 10.1016/S1470-2045(07)70099-2.

16. Corrao G, Bagnardi V, Zambon A, La Vecchia C (2004) A meta-analysis of alcohol consumption and the risk of 15 diseases. Preventive Medicine 38: 613-619. doi: 10.1016/j.ypmed.2003.11.027.

17. Roerecke M, Rehm J (2012) The cardioprotective association of average alcohol consumption and ischaemic heart disease: a systematic review and meta-analysis. Addiction 107: 1246-1260. 10.1111/j.1360-0443.2012.03780.x.

18. Taylor B, Irving HM, Kanteres F, Room R, Borges G, et al. (2010) The more you drink, the harder you fall: A systematic review and meta-analysis of how acute alcohol consumption and injury or collision risk increase together. Drug and Alcohol Dependence 110: 108-116. doi: 10.1016/j.drugalcdep.2010.02.011.

19. Rehm J, Room R, Taylor B (2008) Method for moderation: measuring lifetime risk of alcohol-attributable mortality as a basis for drinking guidelines. Int J Methods Psychiatr Res 17: 141-151. 10.1002/mpr.259.

20. Møller H, Damm M, Laursen B (2012) Ulykker i Danmark [Injuries in Denmark].

21. Barendregt JJ, Van Oortmarssen GJ, Vos T, Murray CJ (2003) A generic model for the assessment of disease epidemiology: the computational basis of DisMod II. Popul Health Metr 1: 4.

22. Lynge E, Sandegaard JL, Rebolj M (2011) The Danish National Patient Register. Scandinavian Journal of Public Health 39: 30-33.

23. Helweg-Larsen K (2011) The Danish Register of Causes of Death. Scandinavian Journal of Public Health 39: 26-29.

24. WHO (2013) Global Burden of Disease 2010. <http://www> healthmetricsandevaluation org/gbd/ .

25. Hansen AB, Hvidtfeldt UA, Gronbaek M, Becker U, Nielsen AS, et al. (2011) The number of persons with alcohol problems in the Danish population. Scand J Public Health 39: 128-136. 39/2/128 [pii];10.1177/1403494810393556 [doi].

26. Hvidtfeldt U, Hansen A, Grønbæk M, Tolstrup J (2008) Alkoholforbrug i Danmark - Kvantificering og karakteristik af storforbrugere og afhængige [Alcohol consumption in Denmark - quantification and caracteristics of people with harmful consumption and dependece].

27. Dawson DA, Grant BF, Stinson FS, Chou PS, Huang B, et al. (2005) Recovery from DSM-IV alcohol dependence: United States, 2001-2002. Addiction 100: 281-292. ADD964 [pii];10.1111/j.1360-0443.2004.00964.x [doi].

28. Begg S, Vos T, Barker B, Stevenson C, Stanley L, et al. (2007) The burden of disease and injury in Australia 2003.

29. Murray, Christopher J. L. and Lopez, Alan D. (1996) The global burden of disease - a comprehensive assessment of mortality and disability from diseases, injuries, and risk factors in 1990 and projected to 2020. Harvard University Press.

30. Stouthard M, Essink-Bot M, Bonsel G, Barendregt J, Kramers P, et al. (1997) Disability weights for diseases in The Netherlands.

31. Danish Health and Medicines Authority (2012) Sundhedsdata [Health data]. <http://www> sst dk/Indberetning%20og%20statistik/Sundhedsdata aspx .

32. Unal B, Critchley JA, Capewell S (2004) Explaining the Decline in Coronary Heart Disease Mortality in England and Wales Between 1981 and 2000. Circulation 109: 1101-1107.

33. Morgenstern H, Bursic ES (1982) A method for using epidemiologic data to estimate the potential impact of an intervention on the health status of a target population. J Community Health 7: 292-309.

34. Barendregt JJ, Veerman JL (2010) Categorical versus continuous risk factors and the calculation of potential impact fractions. J Epidemiol Community Health 64: 209-212. jech.2009.090274 [pii];10.1136/jech.2009.090274 [doi].

35. ECREPH (The European Centre for Register Based Health Related Population Research) (2011) Register Database - Find information on 35 Danish Registers suitable for health related register research. <http://www> si-folkesundhed dk/Statistik/Registerforskning/Database aspx?lang=en .

36. Rehm J, Taylor B (2012) Deriving Alcohol-Attributable Fractions based on exposure and Relative Risks.

37. Cherpitel CJ (1996) Alcohol in fatal and nonfatal injuries: a comparison of coroner and emergency room data from the same county. Alcohol Clin Exp Res 20: 338-342.

38. Cherpitel CJ (1994) Alcohol and casualties: a comparison of emergency room and coroner data. Alcohol Alcohol 29: 211-218.

39. Sahl Andersen J, De Fine Olivarius N, Krasnik A (2011) The Danish National Health Service Register. Scandinavian Journal of Public Health 39: 34-37.

40. Wallach Kildemoes H, Toft Sørensen H, Hallas J (2011) The Danish National Prescription Registry. Scandinavian Journal of Public Health 39: 38-41.

41. Roerecke M, Rehm J (2011) Ischemic Heart Disease Mortality and Morbidity Rates in Former Drinkers: A Meta-Analysis. American Journal of Epidemiology 173: 245-258.

1. YLD = Years Lost due to Disability. Calculated based on incidence, duration and severity of disease[3]. [↑](#footnote-ref-1)
2. This should not introduce major inaccuracies in our analyses, since unpublished results from the Global Burden of Disease 2010 study show that estimates of pYLD are very alike for the two countries [Personal communication with Theo Vos]. [↑](#footnote-ref-2)
3. The Municipality of Copenhagen, the capital of Denmark, is the largest municipality in the country, with 10% of the Danish population. [↑](#footnote-ref-3)
