# Supplementary material for: Cost-Effectiveness of Preventive Interventions to Reduce Alcohol Consumption in Denmark
Source: PLoS One. 2014 Feb 5;9(2):e88041. doi: 10.1371/journal.pone.0088041 (PMC3914889; doi:10.1371/journal.pone.0088041)
Supplement: Table S1 — Results of sensitivity analysis 1. (DOCX) [file pone.0088041.s003.docx]

Table S1

Results of sensitivity analysis 1

| Table S1. Cost-effectiveness of alcohol interventions for the Danish population aged 16+ (population in 2009: 4.5 million), assuming the risk profile of former drinkers for all abstainers. | | | | | | | | | | | | | | | | |
| --- | --- | --- | --- | --- | --- | --- | --- | --- | --- | --- | --- | --- | --- | --- | --- | --- |
| Intervention | | DALYs prevented^a^ | | | Cost offsets (€ million) | | | Intervention cost (€ million) | | | Net cost (€ million) | | | ICER^b^ (€/DALY) | | |
|  |  | Mean | CI95% low | CI95% high | Mean | CI95% low | CI95% high | Mean | CI95% low | CI95% high | Mean | CI95% low | CI95% high | Mean^c^ | CI95% low | CI95% high |
| 1. | 30% taxation | 1,895 | 1,521 | 2,277 | -9.7 | -12.8 | -7.3 | - | - | - | -9.7 | -12.8 | -7.3 | Dominant | Dominant | Dominant |
| 2. | Minimum legal drinking age | 115 | 87 | 145 | 0.01 | 0.01 | 0.01 | 0.6 | 0.6 | 0.7 | 0.7 | 0.6 | 0.7 | 5,665 | 5,142 | 6,482 |
| 3. | Advertising bans | 2,819 | 2,264 | 3,339 | -16.9 | -21.6 | -12.7 | 0.4 | 0.3 | 0.4 | -16.5 | -21.2 | -12.3 | Dominant | Dominant | Dominant |
| 4. | Reduced retail opening hours | 2,146 | 1,725 | 2,571 | -12.7 | -16.2 | -9.7 | 0.6 | 0.6 | 0.7 | -12.0 | -15.6 | -9.0 | Dominant | Dominant | Dominant |
| 5. | Brief intervention | 382 | 236 | 544 | -2.3 | -3.5 | -1.4 | 2.2 | 1.8 | 2.7 | -0.1 | -1.3 | 1.0 | Dominant | Dominant | 1,776 |
| 6. | Longer intervention | 128 | 76 | 181 | -0.8 | -1.2 | -0.5 | 8.9 | 7.3 | 10.8 | 8.1 | 6.4 | 10.0 | 63,587 | 55,502 | 83,832 |
| ^a^ DALY = disability-adjusted life year. ^b^ICER = incremental cost-effectiveness ratio. ^c^Calculated as ‘ratio of means‘[1] | | | | | | | | | | | | | | | | |

Reference List

1. Stinnett AA, Paltiel AD (1997) Estimating CE ratios under second-order uncertainty: the mean ratio versus the ratio of means. Med Decis Making 17: 483-489.
