# Supplementary material for: Cost-Effectiveness of Preventive Interventions to Reduce Alcohol Consumption in Denmark
Source: PLoS One. 2014 Feb 5;9(2):e88041. doi: 10.1371/journal.pone.0088041 (PMC3914889; doi:10.1371/journal.pone.0088041)
Supplement: Table S2 — Results of sensitivity analysis 2. (DOCX) [file pone.0088041.s004.docx]

Table S2

Results of sensitivity analysis 2

| Table S2. Cost-effectiveness of alcohol interventions for the Danish population aged 16+ (population in 2009: 4.5 million) with a 10-year intervention time frame. | | | | | | | | | | | | | | | | |
| --- | --- | --- | --- | --- | --- | --- | --- | --- | --- | --- | --- | --- | --- | --- | --- | --- |
| Intervention | | DALYs prevented^a^ | | | Cost offsets (€ million) | | | Intervention cost (€ million) | | | Net cost (€ million) | | | ICER^b^ (€/DALY) | | |
|  |  | Mean | CI95% low | CI95% high | Mean | CI95% low | CI95% high | Mean | CI95% low | CI95% high | Mean | CI95% low | CI95% high | Mean^c^ | CI95% low | CI95% high |
| 1. | 30% taxation | 29,884 | 23,834 | 35,720 | -178.9 | -222.8 | -136.5 | - | - | - | -178.9 | -222.8 | -136.5 | Dominant | Dominant | Dominant |
| 2. | Minimum legal drinking age | 1,095 | 830 | 1,370 | 0.07 | 0.05 | 0.10 | 3.8 | 3.2 | 4.6 | 3.9 | 3.2 | 4.6 | 3,577 | 3,900 | 3,391 |
| 3. | Advertising bans | 5,548 | 4,453 | 6,669 | -33.6 | -42.7 | -25.6 | 1.2 | 1.0 | 1.3 | -32.5 | -41.6 | -24.4 | Dominant | Dominant | Dominant |
| 4. | Reduced retail opening hours | 4,841 | 3,926 | 5,776 | -28.8 | -36.8 | -22.0 | 3.6 | 3.0 | 4.3 | -25.2 | -33.2 | -18.5 | Dominant | Dominant | Dominant |
| 5. | Brief intervention | 385 | 229 | 545 | -2.4 | -3.6 | -1.4 | 19.6 | 16.0 | 23.6 | 17.2 | 13.4 | 21.4 | 47,163 | 27,556 | 81,271 |
| 6. | Longer intervention | 130 | 81 | 187 | -0.8 | -1.2 | -0.5 | 78.1 | 63.9 | 93.4 | 77.2 | 63.0 | 92.5 | 621,995 | 389,392 | 1,003,728 |
| ^a^ DALY = disability-adjusted life year. ^b^ICER = incremental cost-effectiveness ratio. ^c^Calculated as ‘ratio of means‘[1] | | | | | | | | | | | | | | | | |

Reference List

1. Stinnett AA, Paltiel AD (1997) Estimating CE ratios under second-order uncertainty: the mean ratio versus the ratio of means. Med Decis Making 17: 483-489.
