# Supplementary material for: Cost-Effectiveness of Preventive Interventions to Reduce Alcohol Consumption in Denmark
Source: PLoS One. 2014 Feb 5;9(2):e88041. doi: 10.1371/journal.pone.0088041 (PMC3914889; doi:10.1371/journal.pone.0088041)
Supplement: Table S3 — Results of sensitivity analysis 3. (DOCX) [file pone.0088041.s005.docx]

Table S3

Results of sensitivity analysis 3

| Table S3. Cost-effectiveness of alcohol interventions for the Danish population aged 16+ (population in 2009: 4.5 million) analysed without disease and case fatality trends. | | | | | | | | | | | | | | | | |
| --- | --- | --- | --- | --- | --- | --- | --- | --- | --- | --- | --- | --- | --- | --- | --- | --- |
| Intervention | | DALYs prevented^a^ | | | Cost offsets (€ million) | | | Intervention cost (€ million) | | | Net cost (€ million) | | | ICER^b^ (€/DALY) | | |
|  |  | Mean | CI95% low | CI95% high | Mean | CI95% low | CI95% high | Mean | CI95% low | CI95% high | Mean | CI95% low | CI95% high | Mean^c^ | CI95% low | CI95% high |
| 1. | 30% taxation | 1,918 | 1,567 | 2,273 | -9.9 | -12.7 | -7.4 | - | - | - | -9.9 | -12.7 | -7.4 | Dominant | Dominant | Dominant |
| 2. | Minimum legal drinking age | 129 | 99 | 161 | 0.01 | 0.01 | 0.01 | 0.6 | 0.6 | 0.7 | 0.7 | 0.6 | 0.7 | 5,080 | 4,652 | 5,707 |
| 3. | Advertising bans | 3,220 | 2,574 | 3,866 | -17.2 | -22.7 | -12.7 | 0.4 | 0.3 | 0.4 | -16.9 | -22.3 | -12.3 | Dominant | Dominant | Dominant |
| 4. | Reduced retail opening hours | 2,438 | 1,964 | 2,949 | -13.0 | -17.1 | -9.7 | 0.6 | 0.6 | 0.7 | -12.4 | -16.5 | -9.1 | Dominant | Dominant | Dominant |
| 5. | Brief intervention | 643 | 393 | 930 | -2.0 | -3.5 | -0.8 | 2.2 | 1.8 | 2.7 | 0.3 | -1.5 | 1.6 | 421 | Dominant | 1,723 |
| 6. | Longer intervention | 216 | 134 | 308 | -0.6 | -1.1 | -0.2 | 9.0 | 7.3 | 10.7 | 8.3 | 6.6 | 10.1 | 38,500 | 32,864 | 49,354 |
| ^a^ DALY = disability-adjusted life year. ^b^ICER = incremental cost-effectiveness ratio. ^c^Calculated as ‘ratio of means‘[1] | | | | | | | | | | | | | | | | |

Reference List

1. Stinnett AA, Paltiel AD (1997) Estimating CE ratios under second-order uncertainty: the mean ratio versus the ratio of means. Med Decis Making 17: 483-489.
